# Supplementary material for: Validating the concept of mutational signatures with isogenic cell models
Source: Nat Commun. 2018 May 1;9:1744. doi: 10.1038/s41467-018-04052-8 (PMC5931590; doi:10.1038/s41467-018-04052-8)
Supplement: Supplementary file 1 — Supplementary Information [file 41467_2018_4052_MOESM1_ESM.pdf]

Supplementary Information

Validating the concept of mutational signatures with isogenic cell models

Xueqing Zou, Michel Owusu, Rebecca Harris, Stephen P. Jackson, Joanna I. Loizou, Serena Nik-Zainal

Supplementary Table

Name	Catalogue Number	Company	Dilution
NUDT1	NB100-109	Novus Biologicals	1:1000
NEIL1	12145-1-AP	Proteintech	1:500
CHK2	05-649	Millipore	1:1000
EXO1	A302-639A	Bethyl Laboratories	1:1000
POLE	GTX132100	GeneTex	1:1000
POLB	ab26343	Abcam	1:1000
POLM	C-1	Santa Cruz	1:500
FANCC	MABC524	Millipore	1:500
MSH6	D60G2	Cell Signaling	1:1000
ACTIN	A5060	SIGMA	1:5000

Supplementary Table 1. Information of catalogue numbers and working dilutions for antibodies.

Supplementary Figures

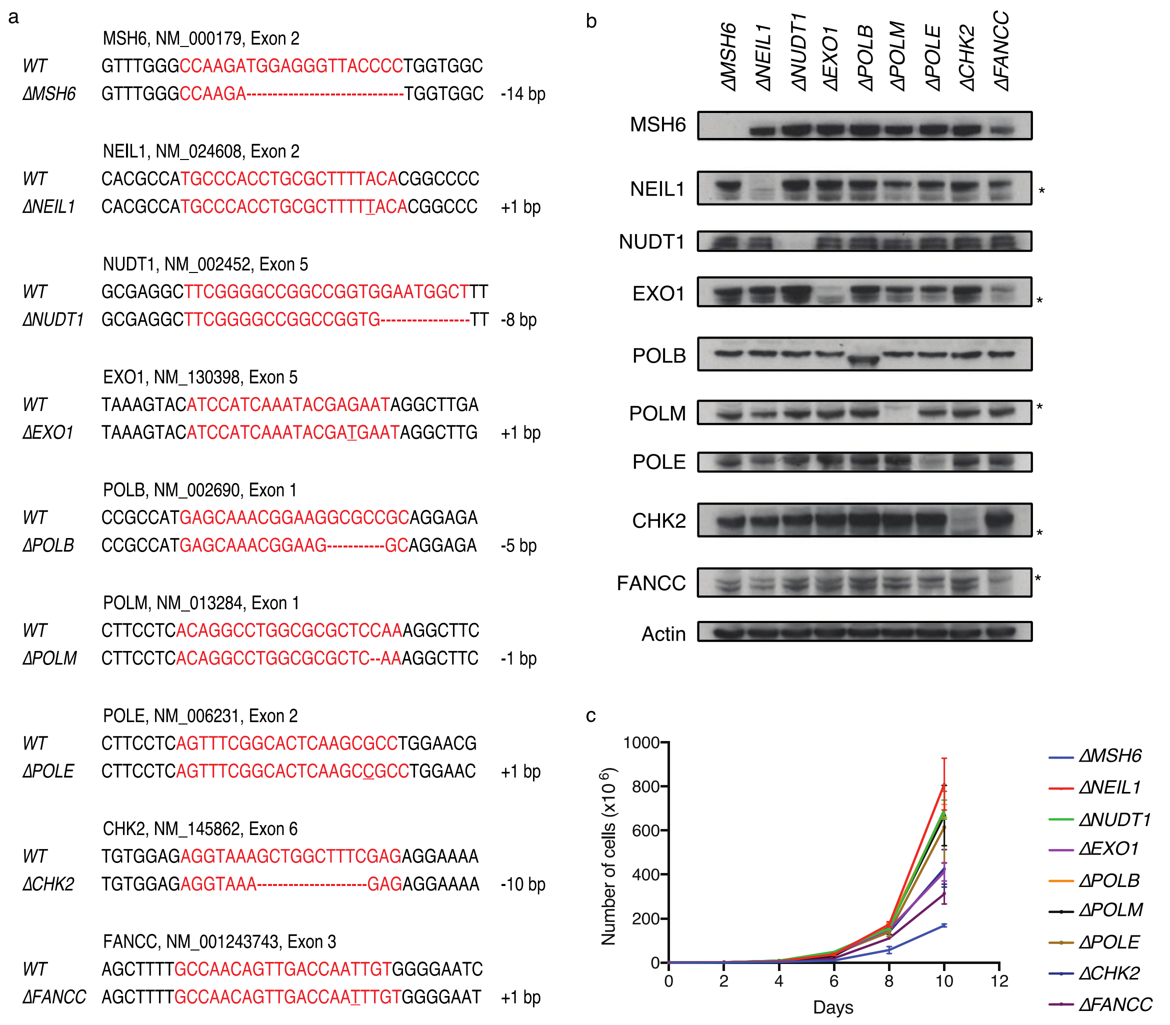

Supplementary Figure 1. Generation and characterisation of CRISPR-Cas9 edited human HAP1 cells. (a) Sanger sequencing confirming CRISPR-Cas9-induced frame-shift mutations in illustrated genes. The red sequence in the wild type (WT) gene corresponds to the guide RNA (gRNA) sequence used. Insertions are marked by an underlined character and deletions by missing sequences (dashes). (b) Immunoblots for expression of MSH6, NEIL1, NUDT1, EXO1, POLB, POLM, POLE, CHK2 and FANCC in CRISPR-Cas9 edited human HAP1 clones. Actin serves as a loading control. "*" denotes a non-specific band. Immunoblot images are shown in Supplementary Figure 9. (c) Proliferation of indicated knockout cell lines over a period of ten days. Living cells were counted every second day using CASY Cell Counter and Analyzer system starting with 0.32 million cells. The plot shows a mean and standard deviation of 3 replicates for each time point. Error bars are defined as standard error of the mean.

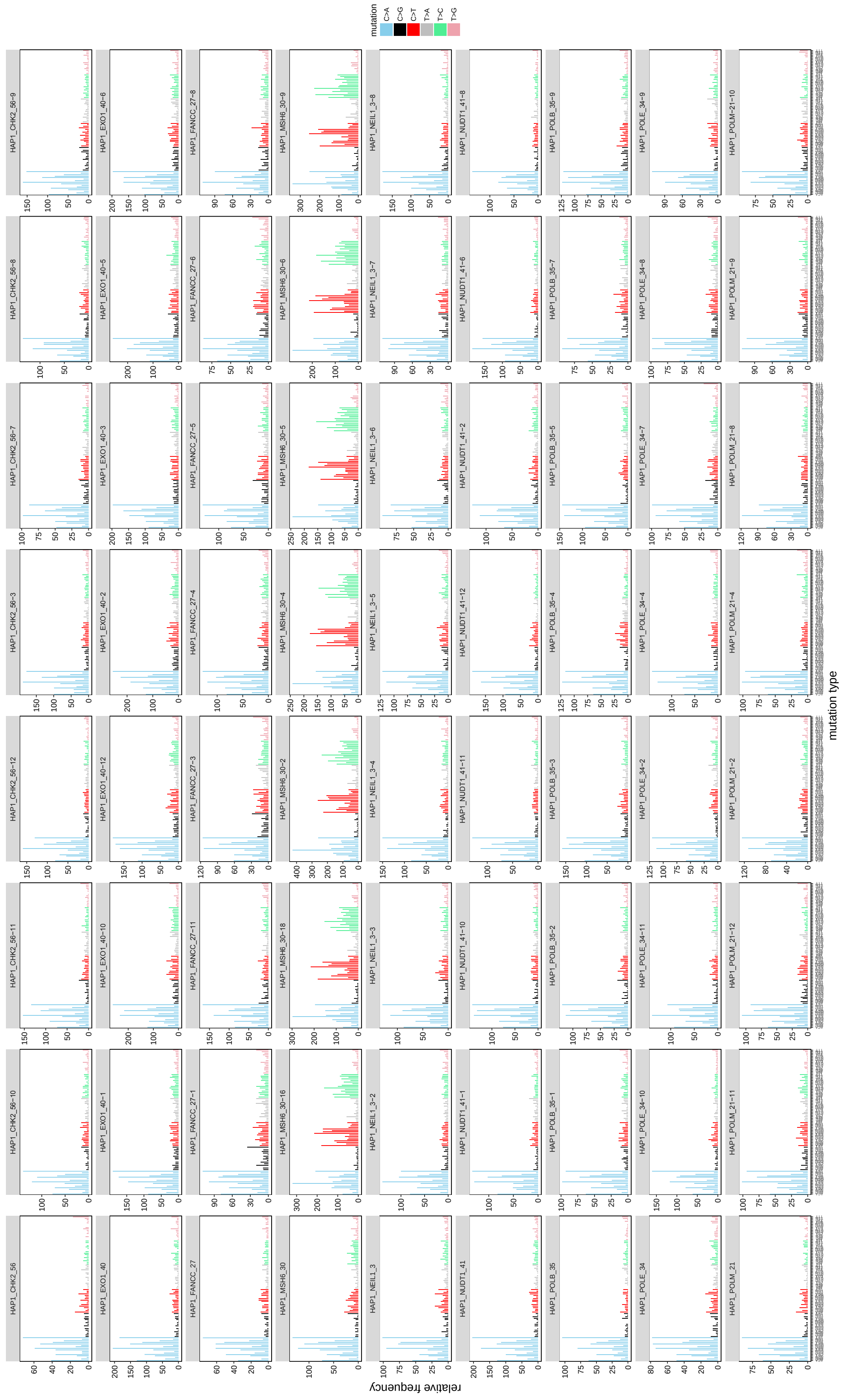
HAP1_CHK2_56
HAP1_CHK2_56−10
HAP1_CHK2_56−11
HAP1_CHK2_56−12
HAP1_CHK2_56−3
HAP1_CHK2_56−7
HAP1_CHK2_56−8
HAP1_CHK2_56−9
HAP1_EXO1_40
HAP1_EXO1_40−1
HAP1_EXO1_40−10
HAP1_EXO1_40−12
HAP1_EXO1_40−2
HAP1_EXO1_40−3
HAP1_EXO1_40−5
HAP1_EXO1_40−6
HAP1_FANCC_27
HAP1_FANCC_27−1
HAP1_FANCC_27−11
HAP1_FANCC_27−3
HAP1_FANCC_27−4
HAP1_FANCC_27−5
HAP1_FANCC_27−6
HAP1_FANCC_27−8
HAP1_MSH6_30
HAP1_MSH6_30−16
HAP1_MSH6_30−18
HAP1_MSH6_30−2
HAP1_MSH6_30−4
HAP1_MSH6_30−5
HAP1_MSH6_30−6
HAP1_MSH6_30−9
HAP1_NEIL1_3
HAP1_NEIL1_3−2
HAP1_NEIL1_3−3
HAP1_NEIL1_3−4
HAP1_NEIL1_3−5
HAP1_NEIL1_3−6
HAP1_NEIL1_3−7
HAP1_NEIL1_3−8
HAP1_NUDT1_41
HAP1_NUDT1_41−1
HAP1_NUDT1_41−10
HAP1_NUDT1_41−11
HAP1_NUDT1_41−12
HAP1_NUDT1_41−2
HAP1_NUDT1_41−6
HAP1_NUDT1_41−8
HAP1_POLB_35
HAP1_POLB_35−1
HAP1_POLB_35−2
HAP1_POLB_35−3
HAP1_POLB_35−4
HAP1_POLB_35−5
HAP1_POLB_35−7
HAP1_POLB_35−9
HAP1_POLE_34
HAP1_POLE_34−10
HAP1_POLE_34−11
HAP1_POLE_34−2
HAP1_POLE_34−4
HAP1_POLE_34−7
HAP1_POLE_34−8
HAP1_POLE_34−9
HAP1_POLM_21
HAP1_POLM_21−11
HAP1_POLM_21−12
HAP1_POLM_21−2
HAP1_POLM_21−4
HAP1_POLM_21−8
HAP1_POLM_21−9
HAP1_POLM−21−10
relative frequency
mutation type
mutation
C>A
C>G
C>T
T>A
T>C
T>G

Supplementary Figure 2. 96-element spectra of substitutions of all parental clones (first column) and subclones. A strong and consistent background signature comprising C>A mutations can be seen for all samples.

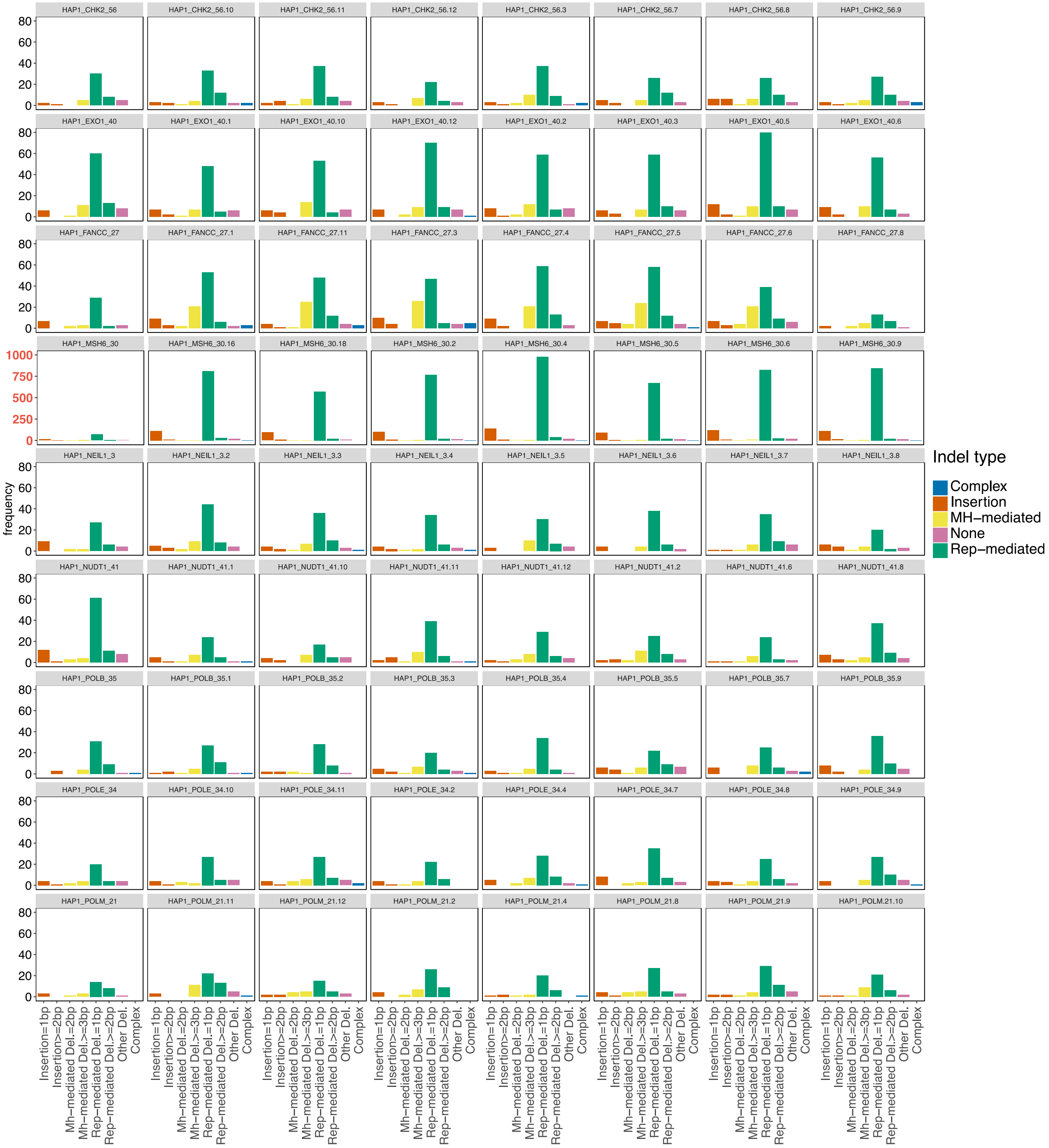

Supplementary Figure 3. 8-element spectra of indels of all parental clones (first column) and subclones. Similar to substitutions, a strong and consistent indel background is observed in all samples.

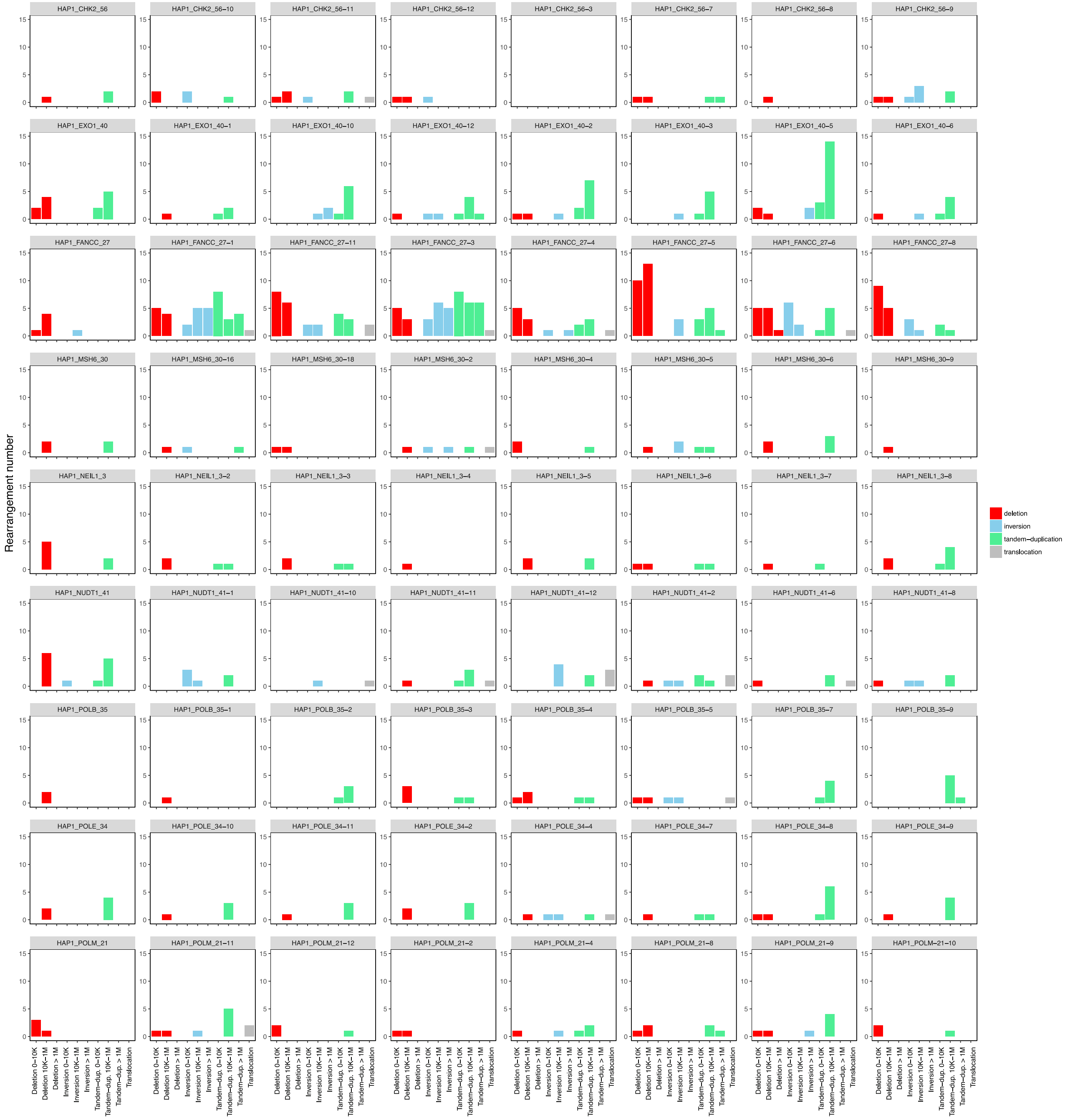

Supplementary Figure 4. 10-element spectra of rearrangements of all parental clones (first column) and subclones. Due to a low number of rearrangements, the profile of rearrangements of each sample is sparser than the profile of indels/substitutions of each sample.

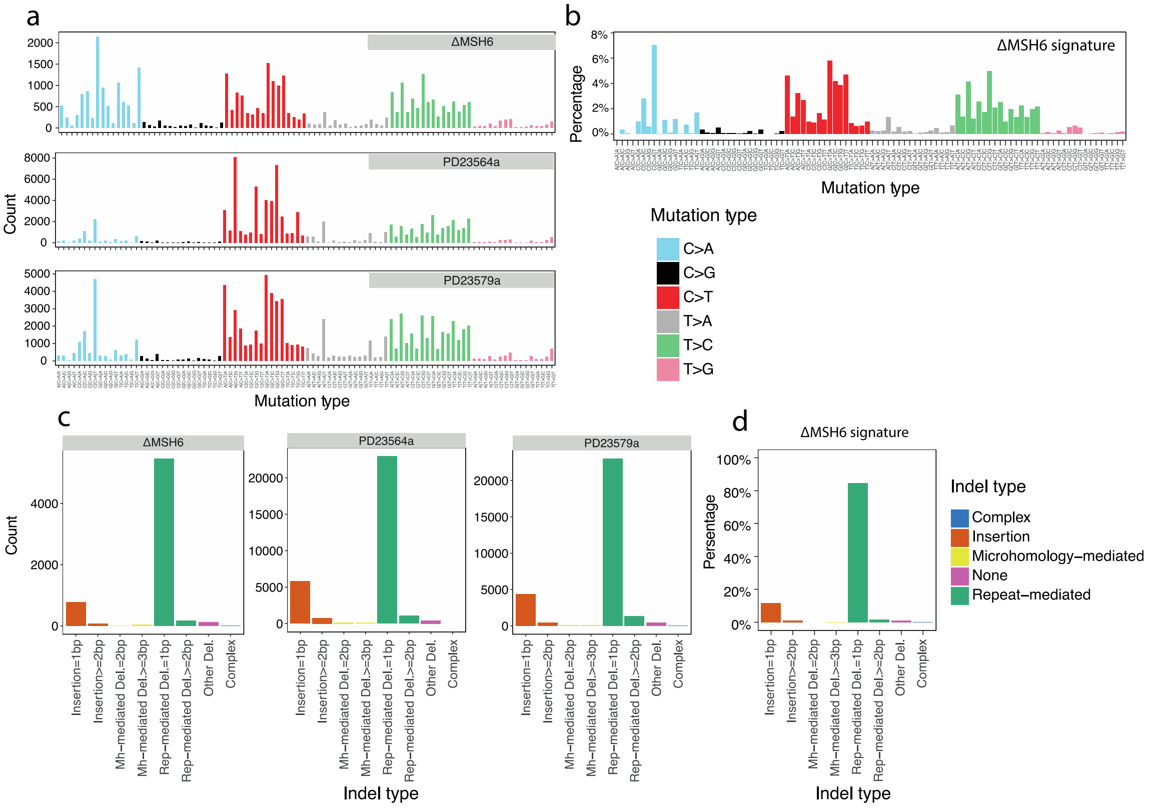

Supplementary Figure 5. Comparison of experimentally generated signatures in mismatch repair (MMR) gene *MSH6* knockouts and mutational signatures in MMR-deficient cancers. (a) 96-element substitution spectra of *MSH6* knockouts (seven subclones were aggregated) and two breast cancers with MMR deficiency, PD23564 and PD23579. (b) Substitution signatures associated with knockout of *MSH6*. (c) 8-element indel spectra of *MSH6* knockouts (seven subclones were aggregated), PD23564 and PD23579. (d) Indel signatures associated with knockout of *MSH6*. Overarching substitution and indel whole genome profiles of MMR-deficient samples are very similar to that of the *MSH6* knockout, particularly the individual substitution and indel profiles.

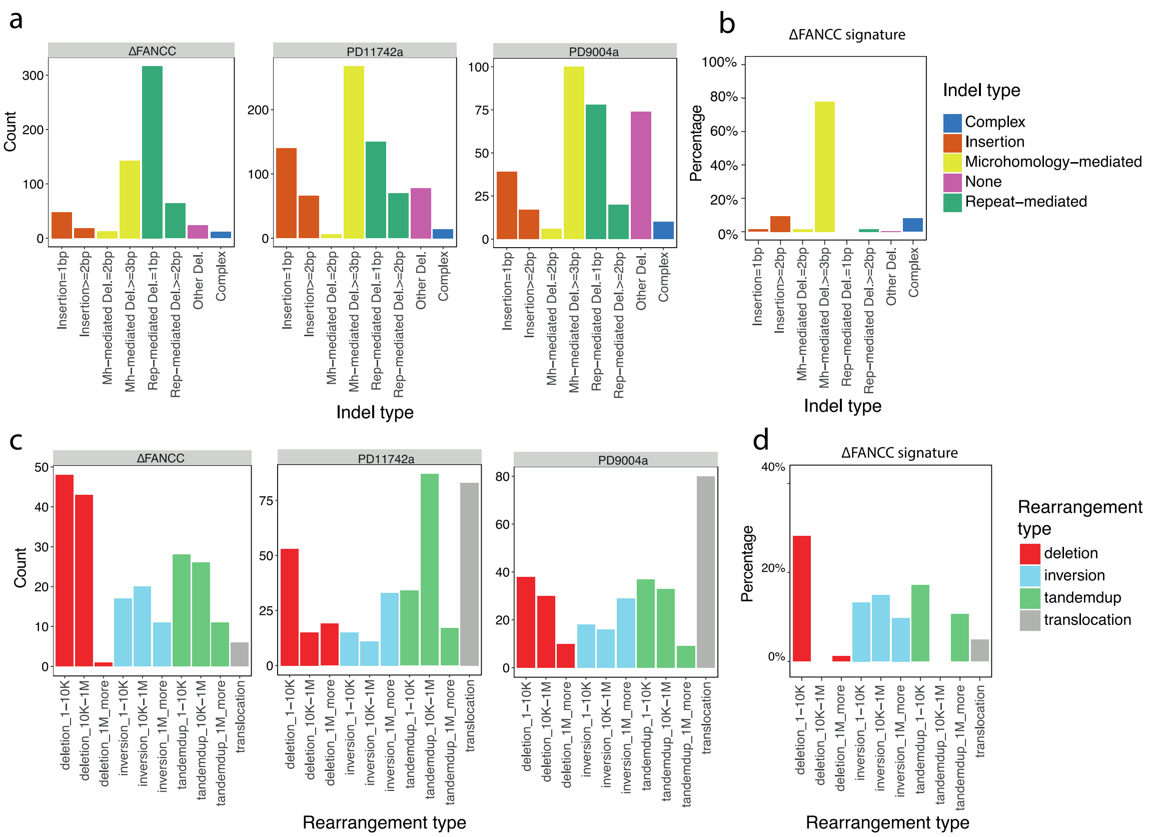

Supplementary Figure 6. Comparison of experimentally generated signatures in DNA cross-link repair via homologous recombination (HR) gene *FANCC* knockouts and cancer-derived mutational signatures in HR-deficient *BRCA1*-null samples. (a) 8-element indel spectra of *FANCC* knockouts (seven subclones were aggregated) and two *BRCA1*-null breast cancers associated with HR deficiency, PD11742 and PD9004. (b) Indel signatures associated with *FANCC* knockouts. (c) 10-element rearrangement spectra of *FANCC* knockouts (seven subclones were aggregated), PD11742 and PD9004. (d) Rearrangement signature associated with *FANCC* knockouts. Indel and rearrangement profiles of *BRCA1*-null samples are very similar to the *FANCC* knockout: microhomology-mediated deletions and a high number of rearrangements (1-10Kb tandem-duplications and 1-10Kb deletions).

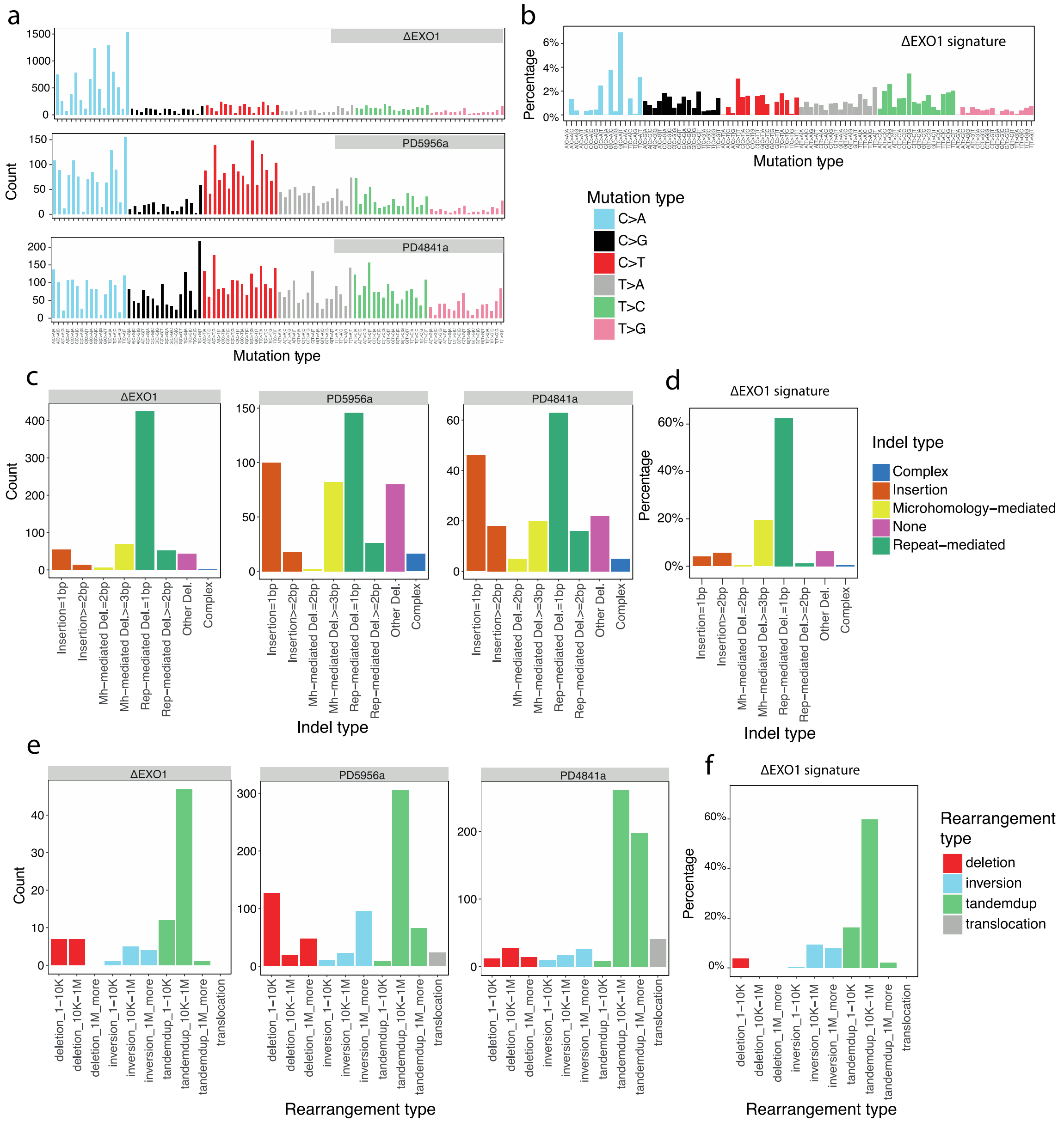

Supplementary Figure 7. Comparison of experimentally-generated signatures in *EXO1* knockouts (*EXO1* is involved in both HR and MMR pathways) and cancer-derived mutational signatures in HR-deficient but *BRCA1*-intact samples. (a) 96-element substitution spectra of *EXO1* knockouts (seven subclones were aggregated) and two *BRCA1*-intact breast cancers with HR deficiency, PD5956 and PD4841. (b) Substitution signatures associated with *EXO1* knockouts. (c) 8-element indel spectra of *EXO1* knockouts (seven subclones were aggregated), PD5956 and PD4841. (d) Indel signature associated with *EXO1* knockouts. (e) 10-element rearrangement spectra of *EXO1* knockouts (seven subclones were aggregated), PD5956 and PD4841. (f) Rearrangement signature associated with *EXO1* knockouts. Indel and rearrangement profiles of *BRCA1*-intact samples are very similar to the *EXO1* knockout: featuring a high number of repeat-mediated deletions and long (>100kb) tandem duplication rearrangements.

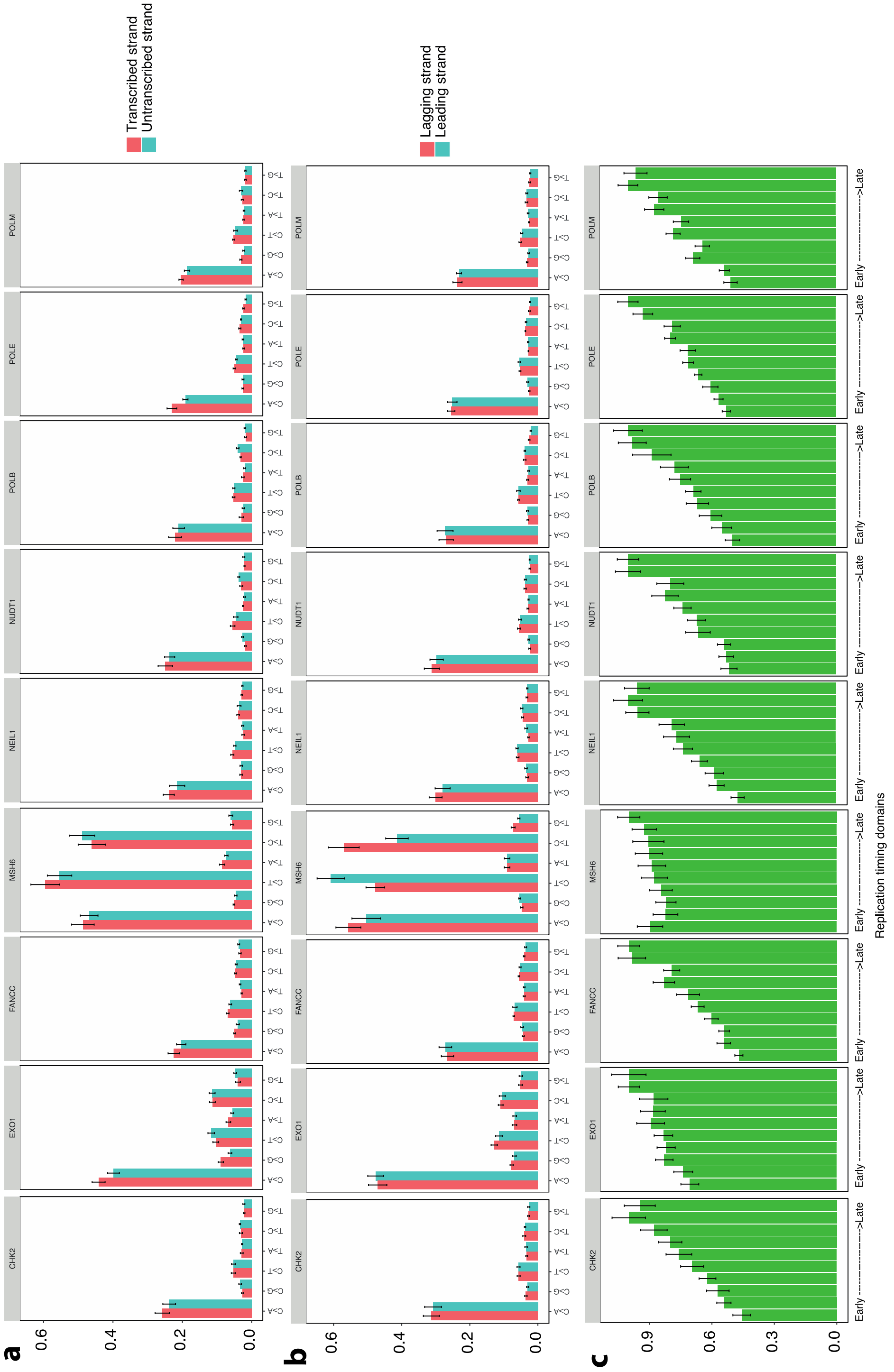

Supplementary Figure 8. The topography of experimentally-generated mutations of nine knockout genes. (a) Histograms exploring transcriptional strand asymmetry. (b) Histograms exploring replication strand asymmetry. (c) Distribution of normalized mutation density across the replication timing domains. Error bars are defined as standard error of the mean.

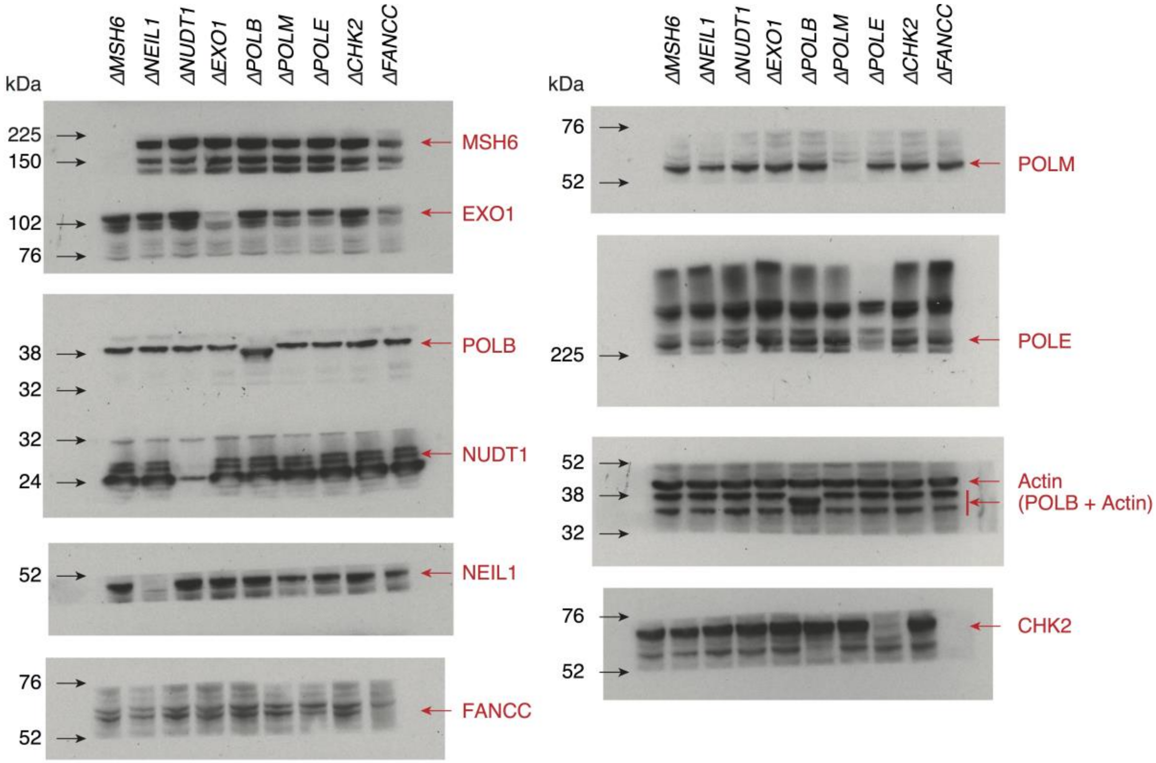

Supplementary Figure 9. Uncropped Immunoblots from Supplementary Figure 1b.
