# Supplementary material for: Validating the concept of mutational signatures with isogenic cell models
Source: Nat Commun. 2018 May 1;9:1744. doi: 10.1038/s41467-018-04052-8 (PMC5931590; doi:10.1038/s41467-018-04052-8)
Supplement: Supplementary file 3 — Description of Additional Supplementary Files [file 41467_2018_4052_MOESM3_ESM.pdf]

**Description of Additional Supplementary Files**

Supplementary Data 1. Confirmation of frameshifting indels in target genes.

Supplementary Data 2. Ranked-list of potential off-target sites of the relevant guide RNA sequences generated by COSMID.

Supplementary Data 3. DNA repair genes tested in off-target analysis.

Supplementary Data 4. Mutation rate of seven knockout signatures.

Supplementary Data 5. Sequence coverage statistics for all parental clones and subclones.

Supplementary Data 6. Summary of de novo somatic mutations (substitutions, indels and rearrangements) in all parental clones and subclones.
